# A Case of Modified McLaughlin Procedure in Neglected Posterior Shoulder Fracture Dislocation

**DOI:** 10.7759/cureus.34356

**Published:** 2023-01-29

**Authors:** Ishan Shevate, Rahul Salunkhe, Ketan Kulkarni, Kishore Janapamala, Ashwin Deshmukh

**Affiliations:** 1 Orthopaedics, Dr. D. Y. Patil Medical College, Hospital and Research Centre, Pune, IND

**Keywords:** shoulder dislocation, mclaughlin procedure, hill-sachs lesion, posterior dislocation of the shoulder, posterior locked shoulder dislocation, reverse hill-sachs lesion, neglected posterior shoulder fracture dislocation, modified mclaughlin procedure

## Abstract

A Hill-Sachs lesion, a posterolateral bony defect of the proximal humerus, occurs when the humerus head collides with the anterior region of the glenoid during an anterior shoulder dislocation. A posteriorly dislocated shoulder may cause a reverse Hill-Sachs lesion, which is a deficiency on the anteromedial part of the humeral head due to impaction. Avascular necrosis could result from this lesion if detection and repair are not carried out. The subscapularis tendon is separated from the smaller tuberosity using an open technique in the original McLaughlin procedure, which was initially described in 1952. In neglected cases of patients undergoing surgery after three weeks, there is no commonly accepted standard of care. Glenohumeral joint stabilization and early and full functional recovery are the two objectives of the procedure. This case report describes a modified McLaughlin surgery where the subscapularis tendon and lesser tuberosity are transferred to the reverse Hill-Sachs defect for stability. The clinical significance of our case report is that it accentuates the role of early detection and appropriate management of reverse Hill-Sachs lesion, which is often overlooked and missed in a case of posterior shoulder dislocation. The use of the modified McLaughlin procedure not only covers the defect with a bone chunk and the subscapularis tendon transfer over the head of the humerus but the stable fixation with the anchor and cannulated cancellous screw helps in early rehabilitation of the shoulder joint.

## Introduction

The posterior shoulder dislocation accounts for 3-5% of all different types of shoulder dislocations [1]. About one in every 100 cases of shoulder dislocation involves a fracture of the proximal humerus [2]. Neer et al. have reported nine cases out of the 15,000 shoulder injury cases having posterior fracture-dislocation [3]. Dislocation of the posterior shoulder can happen following an injury, a seizure, or an electric shock. A posterior shoulder fracture and dislocation may result from the axial force on an arm that is flexed, abducted, and internally rotated. They are classified into atraumatic (caused by the intense muscle spasms that occur after an electric shock or an epileptic seizure) and traumatic (high-velocity injuries like motor vehicle accidents) [4]. A posterior shoulder dislocation results in an impaction fracture, often referred to as a reverse Hill-Sachs defect, on the anteromedial surface of the humeral head. This defect of reverse Hill-Sachs is commonly missed or misdiagnosed in almost 50-79% of patients. Such a lesion should be suspected in patients having a gross restriction of external rotation and a notable eminence of the humeral head in the posterior region of the shoulder along with a more palpable coracoid process [4]. Reverse Bankart lesions may be present along with posterior shoulder dislocations suggestive of a tear of the posteroinferior part of the glenoid labrum and the posterior capsular periosteum being avulsed, resulting in laxity of the glenohumeral ligament of the inferior aspect.

On an anteroposterior (AP) radiograph of the shoulder, there is a half-moon appearance due to the over-imposition of part of the humeral head over the glenoid. A lightbulb sign seen in posterior shoulder dislocation is due to the internal rotation of the humeral head in the AP view [5]. Computed tomography (CT) is utilized to confirm and quantify the articular surface affected by the humeral head over the anteromedial aspect. MRI can be used to reveal the labrum or cuff tears, along with other associated soft tissue injuries.

For the management of such injuries, alternative surgical procedures include arthroplasty, open reduction with internal fixation, and close reduction and pinning. Open reduction and fixation of such fracture dislocations have become the preferred mode of treatment due to the lower costs and related complications. By transferring subscapularis to the lesion and filling the lesion, McLaughlin had established a strategy for the treatment of the reverse Hill-Sachs lesion. Hawkins et al. proposed the modification to the McLaughlin surgery, which involves transferring and fixing the subscapularis tendon and a portion of the lesser tuberosity bone onto the reverse Hill-Sachs defect to stabilize it [5,6]. In a patient with posterior shoulder fracture-dislocation, the modified McLaughlin procedure aims to stabilize the glenohumeral joint and promote an early and complete functional recovery [7]. The size of the defect over the humeral head, the time period since the incident trauma, and the degree of instability influence the treatment options. The primary objective is to preserve the humeral head's structure, while also preserving the postoperative shoulder range of motion [8-11].

Due to the lower costs and associated difficulties, open reduction fixation of these fracture dislocations has emerged as the favored treatment approach. The goal is to restore the humeral head's anatomy, structural integrity, and postoperative shoulder range of movement [12].

## Case presentation

**Figure 1:**
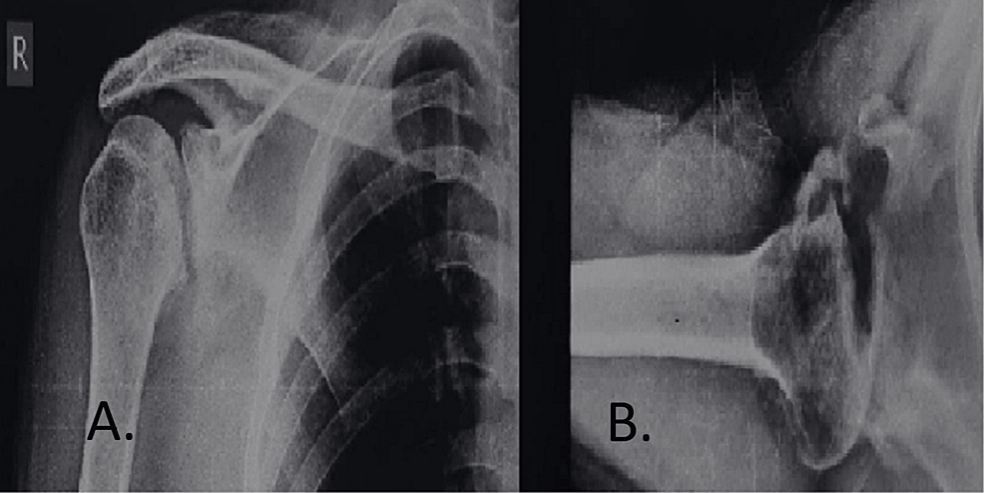
Preoperative X-ray of the patient's right shoulder depicting the anteromedial defect over the head of the humerus. (A) Anteroposterior view. (B) Axillary view.

**Figure 2:**
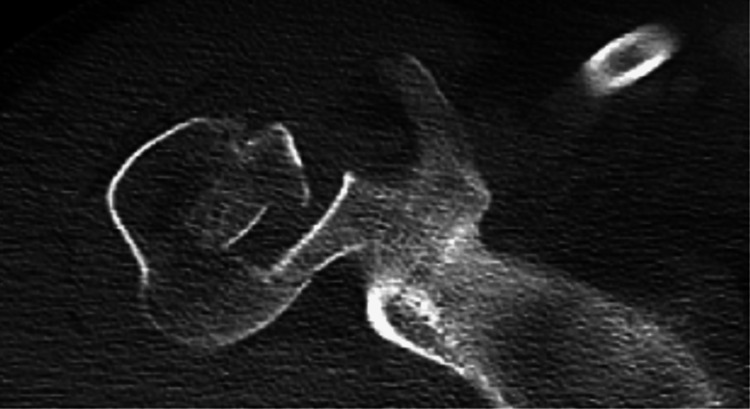
CT scan depicts the extent of the defect.

A 26-year-old male came with complaints of pain over the right shoulder along with restricted shoulder movements for one month following a high-velocity motor vehicle accident posterior shoulder dislocation. Wasting was noted of the deltoid muscle over the right shoulder. The range of movement was minimal and painfully restricted. Gross restriction of internal rotation was noted. On the preoperative AP and axillary X-rays of the right shoulder, a defect was seen on the anteromedial aspect of the humerus (Figure 1) and the CT scan showed the posterior shoulder fracture dislocation. It also helped in quantifying the bone defect (Figure 2) showing impaction fracture involving >40% of the humeral head.

Consent of the patient was taken in view of publication and preoperative counseling was done. The patient underwent the modiﬁed McLaughlin procedure involving the transfer of subscapularis and lesser tubercle over the defect. The patient was operated on in a beach-chair position with a bolster kept under the right scapula to make the right shoulder elevate for an easier approach and also allow ease of manipulation needed intra-operatively.

**Figure 3:**
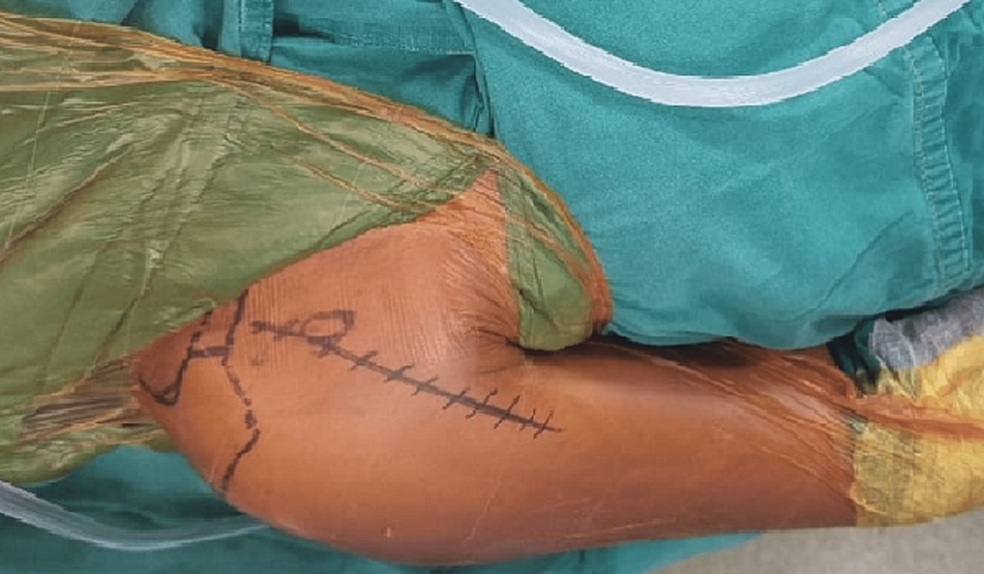
Patient positioning and surgical site preparation.

Scrubbing, painting, draping, and marking of the surgical site incision were done (Figure 3) from the distal to the lateral edge of the clavicle and just lateral to the coracoid process. After incising the skin and superﬁcial fascia, the cephalic vein was identiﬁed and retracted medially.

**Figure 4:**
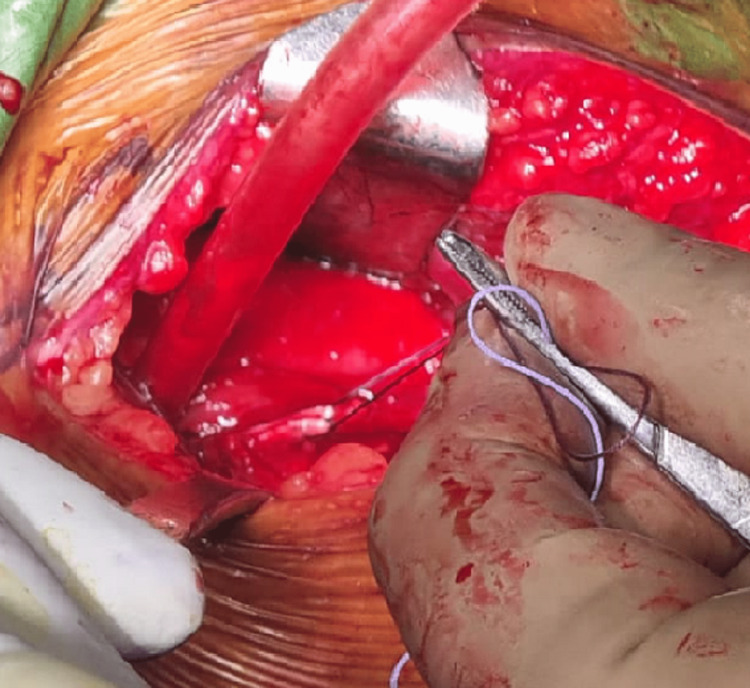
Sutures through the subscapularis tendon.

The deltopectoral interval was identified and then extended to expose the clavipectoral fascia. After exposure, a frayed and thinned-out tendon of the long head of the biceps was noted, for which a tenotomy was done (Figure 4).

**Figure 5:**
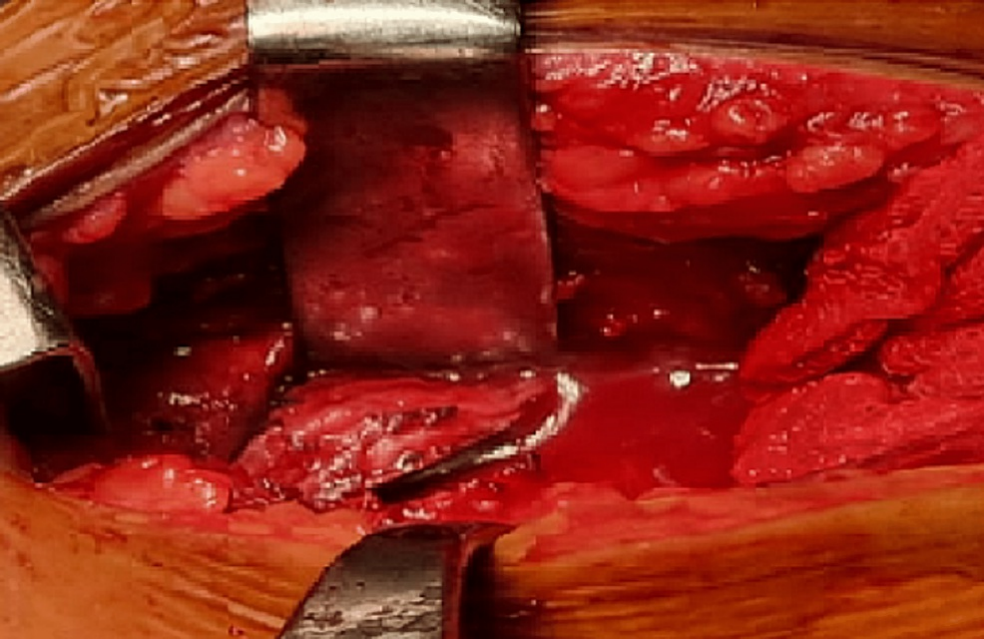
Osteotomized lesser tuberosity bone chunk.

The subscapularis tendon was dissected and reﬂected to expose the joint, and part of the lesser tubercle was osteotomized to create a bone chuck, which was reflected to expose the bony defect beneath the lesser tuberosity (Figure 5).

**Figure 6:**
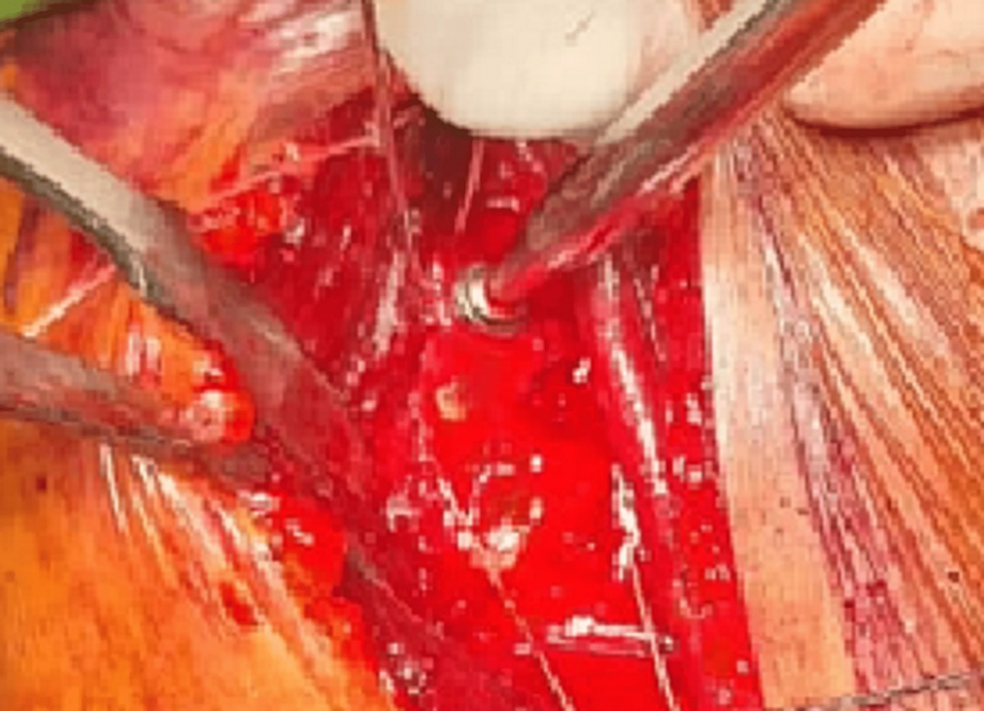
Lesser tuberosity bone chunk fixed with a cannulated cancellous screw.

The tendon of the subscapularis was released superiorly through the interval of the rotator cuff. Two 2.7-mm single-loaded suture anchors were impacted in the superior and inferior part of the defect of the reverse Hill-Sachs lesion over the anteromedial aspect of the head of the humerus after debridement of the area. The suture limbs on the medial side were passed further medial to a lesser tuberosity bone chunk and from the substance of the subscapularis tendon about 5-10 mm from its insertion. Two drill holes were made through the lesser tuberosity with 2 mm Kirschner wire to enable the passage of the opposite suture limbs through the drill holes to hold the ﬁxation, and later the sutures were tied down, snugly pulling the lesser tuberosity along with the subscapularis tendon onto the defect. Another guide wire was passed through the fragment beside the Kirschner wire. A measured partially threaded, cannulated screw with a washer was placed over the guide wire to achieve compression of the fragment of lesser tuberosity onto the humeral defect (Figure 6).

The sutures were tied over the subscapularis and lesser tuberosity, allowing enhanced ﬁxation of the avulsed bony fragment. Glenohumeral joint reduction and screw placement was conﬁrmed by intraoperative ﬂuoroscopy. A normal range of movement of the shoulder joint was visualized.

**Figure 7:**
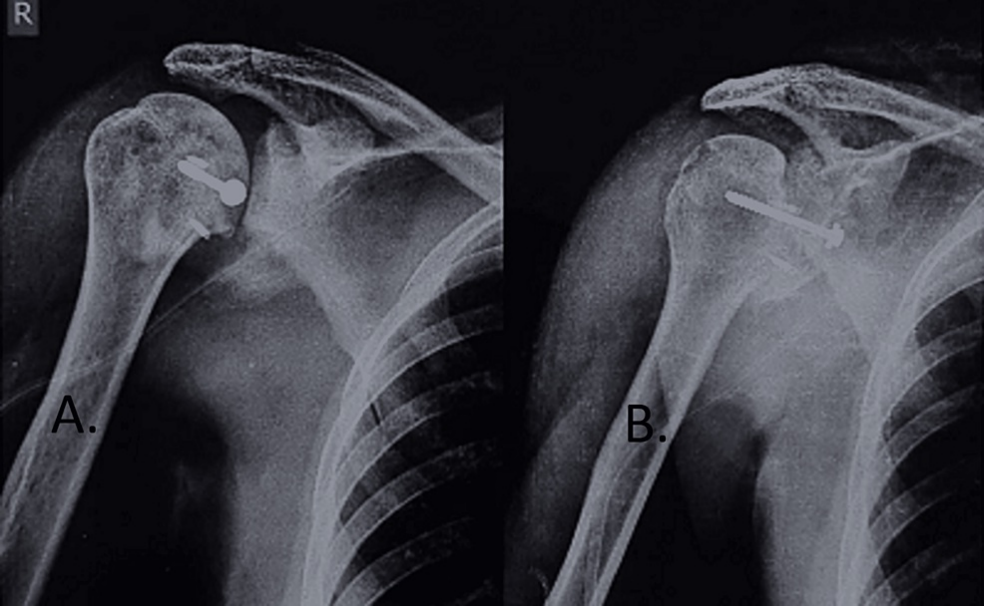
Postoperative X-ray of the patient with the bone chunk over the defect and implant in situ. (A) Anteroposterior view. (B) Lateral view of the proximal humerus.

An interrupted nonabsorbable suture was used for rotator interval closure. Subcutaneous tissue and skin were closed in layers over a suction drain. Figure 7 showed the immediate postoperative X-ray where the defect over the anteromedial aspect of the humerus is covered with the lesser tuberosity bone chunk and with implants in situ.

Postoperative physiotherapy protocol

**Figure 8:**
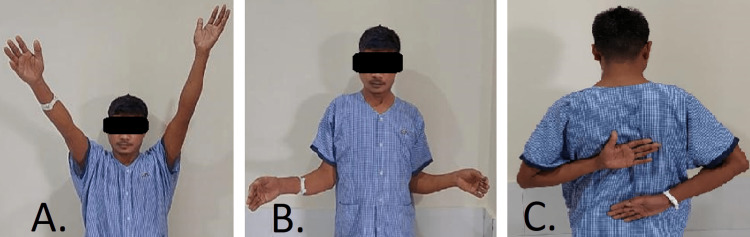
Follow-up after six months with (A) forward flexion of 130 degrees, (B) external rotation of 30 degrees; and (C) internal rotation till L4.

The patient’s shoulder was immobilized using a neutral rotation brace for six weeks, and wrist, hand, and elbow exercises were initiated immediately postoperatively. At four weeks postoperatively, the brace was discontinued and an active assisted shoulder range of movement was initiated. Two months postoperative radiographs showed good glenohumeral reduction along with healing of the defect. The patient had no symptoms and had a pain-free, stable shoulder joint at the time of the last checkup (Figure 8). There had been no recurrence of instability either.

## Discussion

Posterior shoulder dislocation is an uncommon injury. McLaughlin referred to this injury as a diagnostic trap because of how uncommon it is and how frequently it is misdiagnosed. Posterior dislocation of the glenohumeral joint can be suspected with few X-ray signs. In a conventional radiograph of the shoulder, there is a half-moon appearance due to the over-imposition of part of the humeral head over the glenoid. Internal rotation of the humeral head in posterior shoulder dislocation seen on AP view is suggestive of a lightbulb sign [13]. If posterior locked fracture dislocation of the shoulder is suspected, a CT scan is usually done to assess the fracture anatomy and execute preoperative planning. Articular surface involvement of the humerus is assessed particularly with three-dimensional reconstruction and axial and coronal cuts. MRI may be useful to rule out soft tissue and ligament injuries.

Alternative surgical procedures include arthroplasty, open reduction with internal fixation, and close reduction and pinning. Open reduction and fixation of such fracture dislocations have become the preferred mode of treatment due to the lower costs and related complications. The primary objective is to preserve the humeral head's structure while also preserving the postoperative shoulder range of motion [14,15]. The operating surgeon uses these options based on the amount of comminution and impaction. The subscapularis tendon transfer into the defect was suggested by McLaughlin, who is the first surgeon to have recognized the importance of the humeral head impaction fracture associated with shoulder dislocation. A variant of McLaughlin's approach, as reported by Hawkins et al., can be applied to more firmly attach the subscapularis tendon to the defect [8]. Patients had positive results when the subscapularis tendon was transferred and secured into the defect along with the lesser tuberosity. Charalambous et al. suggested fixation of the subscapularis tendon into the defect via suture anchors as an alternative to removing and reattaching the tendon into the lesion [16,17].

Of the previous studies, 25-45% have shown to have excellent outcomes after a modiﬁed McLaughlin procedure in cases of posterior shoulder fracture dislocation. Postoperative rehabilitation protocol is most important for the early rehabilitation of the patient [8].

## Conclusions

AP and axillary X-rays are mandatory in patients presenting with shoulder pain post-trauma with restricted shoulder movements. It is crucial to use a tomographic scan to accurately and quickly diagnose posterior shoulder dislocation to have a better prognosis. The modified McLaughlin surgery had outstanding radiological and clinical results for cases with humeral head defects caused by fracture-dislocation. Postoperative physiotherapy protocols help in regaining complete movements with strength at the earliest. Our study accentuates the role of early detection and appropriate management of reverse Hill-Sachs lesions, which are often overlooked and missed in a case of posterior shoulder dislocation. The use of the modified McLaughlin procedure not only covers the defect with a bone chunk and the subscapularis tendon transfer over the head of the humerus but the stable fixation with anchor sutures and cannulated cancellous screw helps in early rehabilitation of the shoulder joint.
